# Size Matters: What Are the Characteristic Source Areas for Urban Planning Strategies?

**DOI:** 10.1371/journal.pone.0165726

**Published:** 2016-11-10

**Authors:** Zhi-Hua Wang, Chao Fan, Soe W. Myint, Chenghao Wang

**Affiliations:** 1 School of Sustainable Engineering and the Built Environment, Arizona State University, Tempe, AZ, 85287, United States of America; 2 School of Geographical Sciences and Urban Planning, Arizona State University, Tempe, AZ, 85287, United States of America; Potsdam Institute for Climate Impact Research, GERMANY

## Abstract

Urban environmental measurements and observational statistics should reflect the properties generated over an adjacent area of adequate length where homogeneity is usually assumed. The determination of this characteristic source area that gives sufficient representation of the horizontal coverage of a sensing instrument or the fetch of transported quantities is of critical importance to guide the design and implementation of urban landscape planning strategies. In this study, we aim to unify two different methods for estimating source areas, viz. the statistical correlation method commonly used by geographers for landscape fragmentation and the mechanistic footprint model by meteorologists for atmospheric measurements. Good agreement was found in the intercomparison of the estimate of source areas by the two methods, based on 2-m air temperature measurement collected using a network of weather stations. The results can be extended to shed new lights on urban planning strategies, such as the use of urban vegetation for heat mitigation. In general, a sizable patch of landscape is required in order to play an effective role in regulating the local environment, proportional to the height at which stakeholders’ interest is mainly concerned.

## 1. Introduction

Densely built environments are excessively warmer than their rural surroundings, a prominent phenomenon known as the “urban heat island” (UHI) effect [1, 2]. The energetic basis of UHI can be attributed to multiple factors, including modified land surface hydrothermal properties, radiative trapping by buildings, modified aerodynamic roughness, reduced evapotranspiration, and anthropogenic heat release [3–7]. Among these contributors, the use of engineered materials, such as concrete, asphalt, brick, etc. for buildings and pavements, is critical in dictating the UHI effect, given the relatively high thermal storage capacity of these materials. The increase of impervious land cover fractions, together with the rapid urbanization observed in the last few decades [8], has altered the flow patterns of thermal energy in the integrated soil-land-atmosphere continuum [1, 9]. For example, the minimum temperature of Phoenix metropolitan, AZ has experienced an increase of 5.5°C from the late 1940s, and a continuously decreased cooling rate during nighttime due to urban expansion [10, 11].

Among the many adverse environmental impacts of UHI are the significant increase of energy use for cooling of residential and commercial buildings [12–14], and impact on human health with increased heat-related morbidity and mortality [15], especially when exacerbated by heat waves [16]. To alleviate the warming effect induced by impervious engineered pavements, urban vegetation has been widely adopted, common practices including urban lawns, green roofs (also known as “ecoroofs”), shade trees, and urban agriculture [17–19]. Urban vegetation, supplied with adequate soil water availability, is capable of reducing environmental temperature through the cooling effect of evapotranspiration. In addition, vegetated landscapes in built environments also help to improve storm water management and air quality, and to conserve native ecosystems [20–22].

The high spatial heterogeneity associated with the presence of a mixture of a wide variety of landscape types in patches, e.g. pavement, vegetation, open space, etc., is closely related to environmental and ecological patterns of urban areas [3, 23, 24]. Of particular importance to this study, the quantification of urban landscape patterns and its scale dependence is of critical importance in determining source areas of the urban warming effect. Mitigating UHI through vegetation naturally leads to the following question: What is the adequate size of vegetation for reducing air temperatures, for improving human thermal comfort, and/or increasing building energy efficiency? We utilize the multiple endmember spectral mixture analysis (MESMA) to generate fractions of vegetation and impervious surfaces for a sequence of source areas and identified the optimal size for achieving the maximal cooling/warming effect.

Correlation analysis is conducted between land cover fractions derived from remotely sensed images and urban warming measures, i.e. the 2-m air temperature, from ground-based measurements from local meteorological stations. Spatial variability of these statistical correlation coefficients was conventionally used to quantify the effect of different urban land cover features (impervious or vegetated) on air temperature variations [25, 26]. By statistically correlating land use land cover (LULC) types with air temperatures, these indices implicitly encode the relative contribution of different land cover types to urban warming, which coincides with the idea of source areas or “footprint” of urban warming. Interestingly, the concept of source areas has been developed independently in the area of micrometeorology, originated from the description of characteristic scale for vertical mixing of scalars in atmospheric boundary layer (ABL) [27]. The source weight function or footprint function is used to mathematically describe the spatial distribution of surface sources (in this case, urban landscapes and their associated thermal storage) and a measured signal at a height in the surface layer (e.g. 2-m air temperature) [28].

Our objective in this paper is to provide a first attempt to unify the concepts and methodology of correlation analysis and footprint model to quantify the source areas of urban warming. Though developed independently in different scientific branches, and of different mathematical nature (one statistical and the other deterministic and mechanistic), the two methods are speculated to point to the same end in characterizing the impact of different landscapes on elevated urban environmental temperatures. We will use meteorological measurements of air temperatures from the Arizona’s Meteorological (AZMET) network to validate the numerical predictions. This proof of concept will help us to identify characteristic horizontal lengths (fetches) contributing to urban warming as a function of meteorological conditions and atmospheric stability, and to address fundamental questions such as: are there threshold source areas required for implementation of UHI mitigation strategies (urban lawns, green roofs, cool roofs, etc.)? and how does this source area vary as a function of different practical applications in urban planning (improving human thermal comfort, enhancing building energy efficiency, modifying local atmospheric circulation pattern for pollutant dispersion, etc.)? The extension of the combined methodology is expected to broaden our fundamental understanding of the energetic basis and governing mechanisms of urban sustainability in support of practitioners’ urban planning strategies.

## 2. Methodology

### 2.1. Data and study area

Remote sensing provides a valuable tool to quantify, monitor, and predict land cover changes at an unprecedentedly large spatial scale. A Landsat ETM+ image of 30 m spatial resolution was used to generate detailed land cover fraction maps. The image was acquired over the entire Phoenix metropolitan area on April 19, 2000. We selected the study area as our test site because of the wide variety of LULC classes in this region (e.g., residential, commercial, agriculture, lakes, and exposed soil). Six channels, ranging from blue to the short-wave infrared band, were used as the inputs to the MESMA algorithm. We used a pan-sharpened QuickBird data at 60 cm resolution to validate the accuracy of the MESMA output fractions.

**Fig 1:**
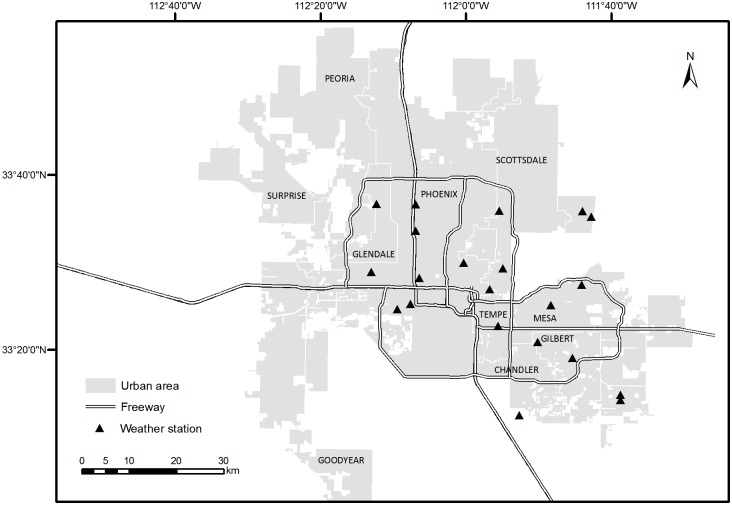
Location of the weather stations in the Phoenix metropolitan area.

The meteorological indicator of urban warming in our study area is the 2-m air temperature in the urban surface layer. The climatic data used in this study was collected from three networks: Arizona Meteorological Network (AZMET), Maricopa County Flood Control District (MCFCD), and Phoenix Realtime Instrumentation for Surface Meteorological Studies (PRISMS) (Fig 1). Collectively, climate data recorded at 21 weather stations were used in our analysis. The dataset encompasses the maximum, minimum, and mean daily air temperature recorded at the 21 weather stations on April 19, 2000, the same date when the satellite image was acquired.

### 2.2. Spatial distribution of vegetation and impervious surfaces

Linear spectral mixture analysis (SMA), which provides sub-pixel land cover (endmember) fractions, is the most commonly used technique of all subpixel approaches. A standard SMA approach employs an invariable set of endmembers to model all pixels. This assumption could potentially lead to significant errors since the number and type of land cover components are highly variable. The endmembers used in SMA are the same for each pixel, regardless of whether the ground cover types represented by the endmembers are present in the pixel. Unusual land cover types, which may not merit their own land cover endmember, may also be poorly modeled by SMA. In addition, because SMA allows only one endmember per material it does not account for the same material with different spectral responses [29]. Roberts et al. [30] introduced multiple endmember spectral mixture analysis (MESMA), an extension of SMA approach that allows the number and type of endmembers to vary for each pixel within an image. MESMA allows more than one endmember in the scene per ground component, and has proven to be effective in identifying vegetation species and land cover type in Southern California chaparral [31]. Hence, we employed MESMA to quantify impervious, soil, vegetation and shade in the Phoenix metropolitan area; the effectiveness of the method was demonstrated in previous studies [24]. The Pearson correlation between the fraction outputs from MESMA and reference data from Quickbird 60 cm resolution data for soil, impervious, and vegetation were 0.8030, 0.8632, and 0.8496 respectively [32].

### 2.3. Correlation analysis

For each weather station, we created a buffer around the station. The buffer (Euclidean) distance ranges from 30 m to 990 m with an increment of 60 m, resulting in a total of 17 geographic extents. For simplicity, two landcover types, viz. impervious and vegetation were used to characterize the land cover around each station, following the treatment in the previous study [25]. We then calculated the average land cover fraction within each buffer based on the distribution maps obtained from the MESMA. This composes the observations on one of our covariates *x*, i.e. the characteristic length of each geographic extent with 60 m increment. The other correlation variable, *y*, was the maximum/minimum/mean daily air temperature collected from the weather stations.

We used the Pearson product-moment correlation coefficient as our measure of the linear association between the *x* and *y* variables. It ranges from -1 to +1, with -1/+1 indicating the perfect negative/positive correlation. Statistically, Pearson’s correlation coefficient is defined as

$$\rho \left(x,y\right)=\frac{\text{cov}\left(x,y\right)}{\sigma_{x}\sigma_{y}},$$ (1)

where cov(*x*, *y*) is the covariance between *x* and *y*, and *σ* is the standard deviation. The covariance between *x* and *y* is given by

cov(x,y)=E{[x−E(x)][y−E(y)]}. (2)

where *E* is the expected value of a given sample (statistical mean). The Pearson’s correlation coefficient was used as an indicator of the linear dependence between the air temperature measured at the weather station and the land cover fraction assessed at a series of spatial extents centered at the station.

### 2.4. Footprint model

There are numerous models, be it analytical, stochastic or mechanistic, in estimating the footprint of scalars and scalar fluxes in the ABL (see. e.g. [28] for a comprehensive review). Here we adopt a widely-used and extensively tested analytical model developed by Kormann and Meixner [33]. In this model, the distributed concentration footprint of a scalar (e.g. temperature, humidity, concentration of pollutants, etc.) mixing along *x* direction (orientated along the wind direction) is given by

$$c(x,z)=\frac{1}{\Gamma (\mu )}\frac{r}{Uz^{1+m}}\frac{\xi^{\mu }}{x^{\mu }}e^{-\xi /x},$$ (3)

where Γ is the gamma function; *r* = 2 + *m—n* is the shape factor with *m* and *n* the exponents of the power laws describing the vertical profiles of wind speed *u* and eddy diffusivity *K*, via *u*(*z*) = *Uz*^*m*^, and *K*(*z*) = *kz*^*n*^ with *k* a constant coefficient in the power law profile; *μ* = (1 + *m*) / *r*; *U* is the constant in power-law profile of the wind velocity; *z* is the height; and *ξ* is the characteristic length scale defined by

$$\xi \left(z\right)=\frac{Uz^{r}}{r^{2}\kappa },$$ (4)

with *κ* the von Karman constant (0.4). The key of the analytical model is to determine the exponents *m* and *n*, by relating the power law to Monin-Obukhov similarity theory (MOST) for the horizontal wind, as

$$m=\frac{z}{u}\frac{du}{dz}=\frac{u_{*}}{u\kappa }\varphi_{m},$$ (5)

and

$$n=\frac{z}{K}\frac{dK}{dz}=\{\begin{array}{cc} \frac{1}{1+5z/L} & \text{for }L>0\\ \frac{1-24z/L}{1-16z/L} & \text{for }L<0 \end{array},$$ (6)

where u_*_ is the friction velocity, *L* is the Obukhov length for measuring dynamic stability of the surface layer (viz. the dimensional height *z*/*L* > 0 for stable atmosphere, *z*/*L* < 0 unstable, and *z*/*L* = 0 neutral), and *φ*_*m*_ is the stability function following the Businger-Dyer relationship [34]:

$$\varphi_{m}=\{\begin{array}{ll} 1+5z/L & \text{for }L>0\\ {\left(1-16z/L\right)}^{-1/4} & \text{for }L<0 \end{array}.$$ (7)

**Fig 2:**
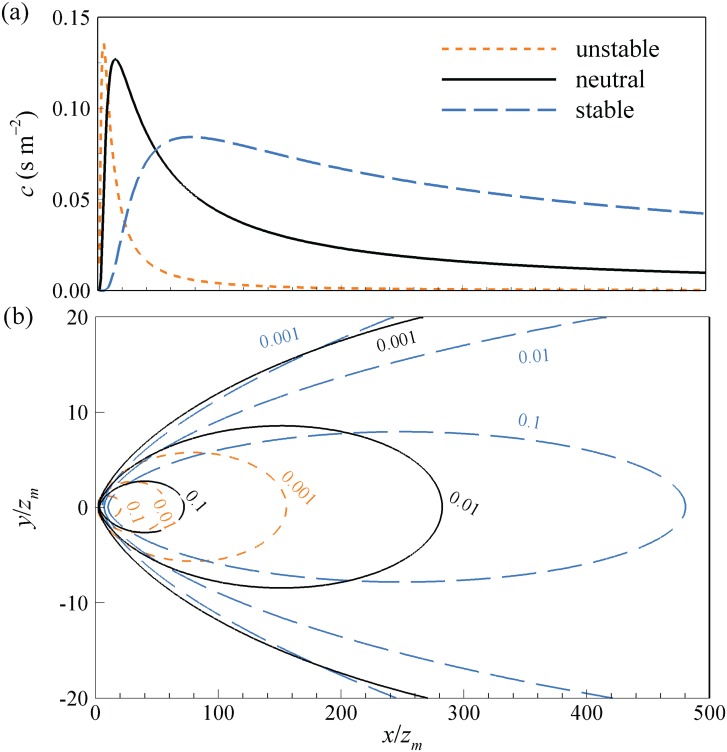
Sample plots of (a) the crosswind integrated footprint function, and (b) isopleths of the footprint with lateral distribution, with the numbers on the isopleths indicating the contour of constant values of crosswind integrated footprint.

In addition, the crosswind distributed concentration footprint is given by,

$$\gamma (x,y,z)=D_{y}c\left(x,z\right)=\frac{1}{\sqrt{2\pi }\sigma }e^{-\frac{y^{2}}{2\sigma^{2}}}\frac{1}{\Gamma (\mu )}\frac{r}{Uz^{1+m}}\frac{\xi^{\mu }}{x^{\mu }}e^{-\xi /x},$$ (8)

where *D*_*y*_ is the crosswind distribution function, and *σ* the dispersion. Fig 2 depicts sample plots of the footprint functions as integrated over the crosswind direction as well as the lateral distributions. It is clear that as the atmospheric instability increases (i.e. more turbulent weather conditions), the concentration of a scalar distributes over a more widely spread area, leading to a larger footprint of the scalar.

## 3. Results and Discussion

**Fig 3:**
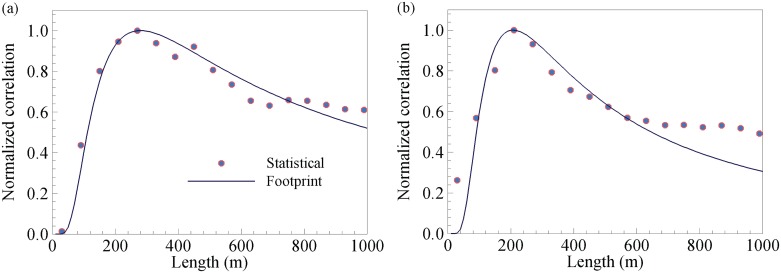
Intercomparison of the normalized correlation between the air temperature and LULC types for (a) vegetation and (b) impervious surfaces at the study site and the normalized footprint, on April 19, 2000. (The average turbulent field data for estimating the footprint are: friction velocity u_*_ = 0.2 m s^-1^, dynamic stability z/*L* = -0.2 unstable, and roughness length *z*_0_ = 0.05 m).

Fig 3 shows the sample result of comparison between the normalized statistical correlations (of the maximum air temperatures recorded by 21 AZMET stations shown in Fig 1 on a typical clear day April 19, 2000 in Phoenix and 17 LULC pixels), and the normalized temperature footprint using the analytical model in Section 2.4. The turbulent and aerodynamic parameters used in estimating the footprint, viz. friction velocity u_*_, Obukhov length *L*, and roughness length *z*_0_, were determined based on measurements from an eddy-covariance tower and a network of weather stations, reported in previous studies [35, 36]. We have conducted similar comparisons between results of the two models under a variety of environmental conditions, including wind directions, weather conditions, atmospheric stability, and other indicators (e.g. minimum or mean temperatures). Most of the comparisons show very similar agreement (not shown here, a complete set of plots of statistical correlation can be found in [25]). This is a pioneering effort linking urban temperature with the contributing source areas at different sizes predicted by two vastly different models: one based on empirical relations (statistical correlation of measurements) and the other on mechanistic turbulence resolution. Both correlation and footprint are normalized by their maximum values (i.e. *y*_normalized_ = *y* /*y*_max_), so to yield the maximum of unity for intercomparison. The agreement between the two methods is reasonably good. Both methods estimate that for the recorded daily maximum air temperature, the maximum contribution is from a source area of around 200 m × 200 m for vegetated and impervious surfaces. Note that the statistical correlations are negative for vegetated surfaces (we plot the absolute values in Fig 3a) and positively for impervious surfaces. This is physical in that urban vegetation tends to mitigate the maximum air temperature by evapotranspirative cooling, whereas the impervious surfaces (mainly engineering pavements) contributes positively to urban warming.

The longer tails observed in the statistical correlations in Fig 3(b), is mainly due to the inhomogeneity of the built landscape. The presence of adjacent built structures (e.g. rooftops) within ~1 km range of the weather stations, which are of similar material properties to impervious surfaces in canyons, likely contribute more to the scalar transport than flat patches due to their elevated heights (so they contribute as stronger “surface” sources). The actual correlation of 2-m air temperature measurement at ground level with its source areas, therefore, exhibits a “folding” or reflection of the scalar contribution due to the neighboring structures, a phenomenon analogous to the aliasing error due to high frequency filtering [37]. On the other hand, note that the analytical footprint model is derived for homogenous surface terrains assuming stationary turbulence, where surface obstacles are treated as homogeneous roughness elements. Thus the contribution from adjacent buildings predicted by the footprint model is smoother and has a faster decay at the tail. This discrepancy between the two methods at the tail within 1 km is likely to be weakened when we move further away from the measurement points, where temperature contribution further decreases, and the impact of surface inhomogeneity will fade as well.

**Fig 4:**
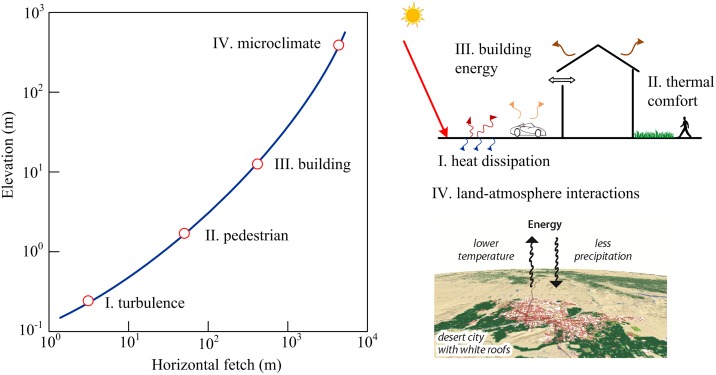
Illustration of the relationship between the measurement height and the characteristic horizontal distance (source area size), with examples at different scale of real applications, including (I) turbulent scale heat dissipation, (II) outdoor thermal comfort, (III) building energy efficiency, and (IV) urban microclimate modeling.

As shown in Fig 3, the maximum footprint *f*_max_ (or maximum statistical correlation *ρ*_max_) occurs at certain source area (upwind in the footprint model) with horizontal dimension *x*_max_. Both are strong functions of the measurement height of the sensing instrument above the land surface. Whereas empirically there is no *a priori* knowledge existed in order to predicted these relations, the analytical footprint model offers a good clue. According to the formulation presented in Section 2.4, it can be readily derived that the maximum laterally-integrated footprint is given by [33] as

$$f_{\text{max}}=\frac{{\left(1+\mu \right)}^{1+\mu }}{\Gamma (\mu )\xi }e^{-1-\mu },$$ (9)

which is found at the upwind distance of

$$x_{\text{max}}=\frac{\xi }{1+\mu },$$ (10)

from the location of the station. The height-dependent footprint distribution is sketched in Fig 4. As the measurement height increases, the characteristic horizontal length increases (usually nonlinearly) as well. Given the agreement between the results of the footprint model, this relation can be readily extended (at least qualitatively) to predict the statistical correlation between measured meteorological variables (not limited to ambient temperatures) and the contributing LULC types. Thus the result of footprint model offers valuable estimates of the adequate sizes of grid-cells (e.g. the coarsest spatial resolution should at least resolve the characteristic length *x*_max_ which represents the maximum correlation) for landscape fragmentation for various geophysical studies.

This source-area-to-measurement-height relation, when closely scrutinized, can have profound implications to urban planning in determining the threshold size of, e.g. vegetation patches for mitigating urban warming. For the particular sites in this study, subjected to local climatological, geophysical, and environmental conditions, the recorded daily maximum temperature using the AZMET stations is most strongly regulated by upwind patches of ~ 100 m in size, negatively by vegetation and positively by impervious surfaces. It can be extended that if urban planners’ primary concerns shift case-by-case, e.g. from enhancing pedestrian’s thermal comfort in street canyons to alleviating environmental thermal stress in urban boundary layer. With the due increase of “height of interest” (here in the sense of the average height at which stakeholders’ interest is mainly concerned), the optimal size of source areas, by estimation, will increase accordingly by a few folds to, e.g. ~ 500–1000 m. This is rather commonplace as we found in hot summer days, patches of lawns and swimming pools of typical characteristic lengths of 10–20 m in residential areas offer comfortable cooling to pedestrians. It also appeals to our physical intuition that a car park paved of asphalt can lead to elevated thermal stress as well as building cooling cost in its vicinity, at a height proportional to its size.

This concept is further illustrated schematically in Fig 4, where four urban planning strategies for four applications with distinctive scales are plotted. These include (I) the sub-meter scale (~ 0.1 m, vertical scale, same hereinafter) turbulent transport that dominants the rate of mixing and dissipation, e.g. mixing of pollutant in street canyons or nocturnal cooling of building facades, (II) the pedestrian scale (~ 1 m) thermal states, e.g. the estimate of outdoor human comfort indices, (III) the building scale (~10 m) interactions with the environment, e.g. energy saving by tree shading, and (IV) the atmospheric boundary layer (ABL) scale (~100–1000 m) microclimate, e.g. urban-land-atmosphere interactions or pollutant dispersion over the city. Depending on the actual application that concerns homeowners, practitioners, or resource managers, the corresponding source areas (e.g. the patch size of urban lawns or sizes of landscape fragmentation) need to be carefully selected in order to yield the desirable effect of landscape planning.

## 4. Concluding Remarks

This study presents a pioneering study in estimating the dependence of urban temperature measurement on characteristic horizontal length, viz. the source area, by unifying the statistical correlation method commonly used in geographical science with the analytical footprint model in meteorological and environmental studies. There is a good agreement between numerical predictions of the two methods, based on weather station measurements in a typical clear day in Phoenix Arizona. It is noteworthy that due to the practical limitation in urban meteorological measurements, especially the lack of wide spectrum in measurement height, results of the current study are constrained to the 2-m air temperature as the signal variable of turbulent transport. In addition, the current results are based on a diurnal measurement (24 hours), where the determination of analytical footprint depends heavily upon the site-specific and time-specific meteorological and flow conditions, especially the prevailing wind direction, friction velocity, and atmospheric stability. Nevertheless, if longterm urban climatology of various cities is considered in real applications, the result of the current study can be readily extended or even simplified. For example, for a design of inter-city transportation network and its impact on regional climate, the prevailing wind direction is likely disappear when large geographic variability is covered, and an directional average of footprint estimate over 360° in the designated region may suffice. Hence, the results of this study can be extended to guide the design of various urban planning strategies, as well as to achieve optimal implementation of urban infrastructure where multiple applications with different scale need to be considered for trade-offs.
